# Thermal biology of flight in a butterfly: genotype, flight metabolism, and environmental conditions

**DOI:** 10.1002/ece3.1758

**Published:** 2015-11-10

**Authors:** Anniina L. K. Mattila

**Affiliations:** ^1^ Metapopulation Research CentreDepartment of BiosciencesUniversity of HelsinkiFI‐00014HelsinkiFinland

**Keywords:** Butterfly flight, dispersal evolution, dispersal modeling, *Flightin*, insect flight, *Pgi*, sex difference, thermal tolerance, thermoregulation

## Abstract

Knowledge of the effects of thermal conditions on animal movement and dispersal is necessary for a mechanistic understanding of the consequences of climate change and habitat fragmentation. In particular, the flight of ectothermic insects such as small butterflies is greatly influenced by ambient temperature. Here, variation in body temperature during flight is investigated in an ecological model species, the Glanville fritillary butterfly (*Melitaea cinxia*). Attention is paid on the effects of flight metabolism, genotypes at candidate loci, and environmental conditions. Measurements were made under a natural range of conditions using infrared thermal imaging. Heating of flight muscles by flight metabolism has been presumed to be negligible in small butterflies. However, the results demonstrate that Glanville fritillary males with high flight metabolic rate maintain elevated body temperature better during flight than males with a low rate of flight metabolism. This effect is likely to have a significant influence on the dispersal performance and fitness of butterflies and demonstrates the possible importance of intraspecific physiological variation on dispersal in other similar ectothermic insects. The results also suggest that individuals having an advantage in low ambient temperatures can be susceptible to overheating at high temperatures. Further, tolerance of high temperatures may be important for flight performance, as indicated by an association of *heat‐shock protein* (*Hsp70*) genotype with flight metabolic rate and body temperature at takeoff. The dynamics of body temperature at flight and factors affecting it also differed significantly between female and male butterflies, indicating that thermal dynamics are governed by different mechanisms in the two sexes. This study contributes to knowledge about factors affecting intraspecific variation in dispersal‐related thermal performance in butterflies and other insects. Such information is needed for predictive models of the evolution of dispersal in the face of habitat fragmentation and climate change.

## Introduction

Loss and fragmentation of natural habitats is the main cause of biodiversity loss and species extinctions (Baillie et al. 2004; IUCN 2014). A key challenge for predicting the biological consequences of habitat fragmentation is to develop mechanistic understanding of individual movements and dispersal, as sufficient dispersal is imperative for population viability in highly fragmented landscapes (Hanski 1999; Ronce 2007). Specific questions in this context include how dispersal is affected by morphological, physiological, and behavioral traits, to what extent, the variation in dispersal rate is governed by genetic versus environmental factors (and genotype × environment interactions), and does natural selection affect relevant traits under changing environmental conditions (Nathan et al. 2008; Clobert et al. 2012). As global temperatures continue to rise and the frequency of thermally extreme conditions increase, knowledge about the influence of ambient temperatures on movements and dispersal is much needed. To address these questions, dissecting dispersal into its components and investigating movements at different spatial scales is a helpful approach (Nathan et al. 2008).

The body temperature of small butterflies and many other flying insects is largely governed by ambient temperature and solar radiation rather than metabolism (i.e., they are ectothermic), which makes them sensitive to changes in thermal conditions (Heinrich 1993; Wickman 2009). Butterflies are especially dependent on flight for most activities during adult life, including foraging, escaping predation, locating mates, searching for host plants, and dispersal (Kingsolver 1983; Saastamoinen and Hanski 2008; Niitepõld et al. 2009; Gibbs 2010). However, insect flight is energetically very costly, and thoracic muscles of flying insects exhibit the highest rates of metabolism known for any locomotor tissue (Dudley 2000; Suarez 2000), exceeding metabolism at rest by up to two orders of magnitude (Kammer and Heinrich 1978). Consequently, the flight of butterflies requires high muscle temperature, between 30 and 38°C in many species (Watt 1968; Heinrich 1993; Wickman 2009), and their activity is strongly affected by thermoregulation.

In temperate climates, the body temperature (*T*_b_) of a butterfly is determined by a balance between heat gained from external heat sources (mostly solar radiation) and heat lost due to convective cooling, which increases with, for example, wind speed (May 1979; Wickman 2009). Butterflies can regulate *T*_b_ behaviorally, and they typically attain suitable *T*_b_ for flight by basking in the sun. Heat is also produced in flight muscles during flight, but the contribution of internal heat production is presumed to be negligible in small butterflies, in which *T*_b_ quickly decreases and approaches ambient air temperature during flight (Shreeve 1984; Heinrich 1986b; Wickman 2009). Small butterflies are therefore forced to land and to bask at regular intervals to regain sufficient *T*_b_ for flight. In contrast, larger species generate enough heat by flight metabolism to stabilize *T*_b_, and species such as *Nymphalis antiopa* and *Colias eurytheme* can continue to fly even in low temperatures (Heinrich 1986a). However, there is no known critical size threshold for such continuous flight, and there can also be inter‐ and intraspecific differences in behavioral thermoregulation strategies (Kemp and Krockenberger 2002, 2004).

Although butterfly thermoregulation is a well‐understood process, and interspecific differences are well studied (Wickman 2009), much less is known about the factors that generate and maintain variation within and among populations (Sinclair et al. 2012). One exception is the effect of morphological traits such as body size and wing and body coloration on preflight heating and cooling during flight, which have been demonstrated in many butterfly species (Watt 1968; Van Dyck and Matthysen 1998; Berwaerts and Van Dyck 2004; Kemp and Krockenberger 2004). These effects can have significant fitness consequences, as being able to fly at low ambient temperatures can enhance fitness through more time being available for reproduction and dispersal (Kingsolver 1983; Saastamoinen and Hanski 2008).

In the Glanville fritillary butterfly (*Melitaea cinxia*; Linnaeus, 1758), allelic variation in the glycolytic gene *phosphoglucose isomerase* (*Pgi*) is associated with many life‐history traits and fitness components (e.g., Hanski and Saccheri 2006; Saastamoinen 2007; Saastamoinen and Hanski 2008; Klemme and Hanski 2009; Saastamoinen et al. 2009). Importantly, many of these associations interact with temperature. In particular, one *Pgi* genotype (SNP *c.331 AC*, which corresponds to the allozyme PGI‐f; Orsini et al. 2009) has superior performance in low ambient temperatures: Individuals with this genotype move more often (Ovaskainen et al. 2008) and longer distances in low ambient temperatures in the field (Niitepõld et al. 2009) than the alternative SNP genotypes. The *AC* heterozygotes also have higher flight metabolic rates at low ambient temperatures (Haag et al. 2005; Niitepõld et al. 2009; Niitepõld 2010), and indeed, flight metabolic rate correlates positively with dispersal rate in the field, explaining up to one‐third of the variation in flight distances (Niitepõld et al. 2009). Finally, the AC heterozygotes have on average higher *T*_b_ as recorded in butterflies caught during flight (Saastamoinen and Hanski 2008). Similar results have been reported for *Pgi* polymorphism in *Colias* butterflies, where a particular *Pgi* genotype is associated with higher flight performance and activity at lower ambient temperatures (Watt et al. 1983, 2003; Watt 1992). Watt et al. (1983) suggested that differences among the genotypes in their ability to fly at low ambient temperatures are due to differences in the kinetic performance of the respective isoforms of the PGI enzyme at different temperatures. In the Glanville fritillary, the *Pgi* SNP *c.331 AA* genotype (allozyme PGI‐d), which is associated with low flight metabolism in standard temperatures, indeed outperforms the other genotypes in high and low ambient temperatures (Niitepõld 2010; Kallioniemi and Hanski 2011). Moreover, the *AA* homozygotes have better tolerance of stressfully high temperatures (Luo et al. 2014), similarly to what has been reported for the Sierra willow beetle *Chrysomela aeneicollis*, in which the thermal stress‐related *heat‐shock protein* (*Hsp*) expression differs between the *Pgi* genotypes (Dahlhoff and Rank 2000; Neargarder et al. 2003; Rank et al. 2007). Saastamoinen and Hanski (2008) suggested that the higher body temperature during flight of the Glanville fritillary *Pgi‐f* genotype in low ambient temperatures could be attributed to either higher takeoff body temperature or differences in flight metabolism. As the flight metabolic rate can differ by as much as 40% between the *Pgi* genotypes (Haag et al. 2005; Niitepõld et al. 2009; Niitepõld 2010), it is feasible that it could influence the thermal dynamics of flight.

Here, these hypotheses are tested by recording the *T*_b_ of butterflies at the time of voluntary takeoff and following a flight bout of known duration. Flight experiments were conducted in a large outdoor population cage, under conditions that closely mimic the environmental conditions experienced by butterflies in the field. Body temperature was measured with IR (infrared) thermal imaging, and the influence of flight metabolic rate and several candidate genes on *T*_b_ was analyzed. The aim of these experiments was to examine intraspecific variation in the thermal dynamics of flight and to study physiological and genetic correlates of this variation at the level of individual flight bouts, the basic component of butterfly movement, and dispersal.

## Materials and Methods

### Study species, sampling, and rearing

The Glanville fritillary butterfly is distributed from West Europe to South Siberia and NW China. In Finland, it occurs at its northern range limit, in the Åland Islands only, where it persists in a large metapopulation of around 4000 habitat patches (small dry meadows with one or both of the host plants *Veronica spicata* and *Plantago lanceolata*) where the turnover rate of local populations is very high (Hanski 1999; Nieminen et al. 2004; Ojanen et al. 2013). In the Åland Islands, the butterfly has a univoltine life cycle, and caterpillars live in sib groups and diapause gregariously (Boggs and Nieminen 2004). Based on mark–release–recapture studies, the mean lifetime dispersal distance is only some hundreds of meters, the longest observed dispersal events are 1–2 km (Kuussaari et al. 1996; Niitepõld et al. 2011), and the longest recorded distances to newly colonized habitats are 4–5 km (van Nouhuys and Hanski 2002). Movement distances and the FMR (rate of flight metabolism) vary greatly among individuals, but FMR is repeatable within an individual (*r* = 0.46–0.91; Niitepõld and Hanski 2013) and significantly heritable (Mattila and Hanski 2014). FMR correlates positively with distances flown and butterfly activity level in the field (Niitepõld et al. 2009), making it a relevant measure of flight capacity in natural conditions.

The butterflies for the present experiments were collected from the field in autumn 2010 as prediapause larvae. The individuals (*n*_females_ = 36, *n*_males_ = 51) originated all from different (87) families in 71 different local populations across the Åland metapopulation. Diapausing larvae were maintained in growth chambers (5°C, 85% relative humidity, RH). Following diapause, larvae were reared individually in common garden conditions (12/12 dark/light 15/28°C) and fed with greenhouse‐grown *P. lanceolata* ad libitum. Adult butterflies were individually marked and maintained in 40 × 50 cm mesh cages under conditions suitable for flight (08–10 light/24°C, 10–15 light/28°C, 15–17 light/24°C, 17–08 dark/18°C, 20% honey–water solution ad libitum). To standardize their activity and nutritional state, butterflies were moved to conditions that discouraged flight activity on the day before the measurement of FMR (dim light, 23°) and provided with water only. In the following day, butterflies were weighed (Mettler‐Toledo XS 105 analytical balance, accuracy 0.01 mg) and their FMR was measured.

### Flight metabolic rate

Flight metabolic rate was measured when the butterfly was 2–3 days old. The age of the butterfly and the time of day of the measurement did not affect FMR (*P *>* *0.05). FMR was measured using flow‐through respirometry (Niitepõld et al. 2009). After acclimatization in a darkened measurement chamber for ~30 min, individuals were stimulated to fly for 7 min in the 1‐L transparent respirometry chamber, through which CO_2_‐free dry air was pumped at the rate of 1.04 L/min. The jar was kept under a ultraviolet light source (UVA, Sylvania Blacklight, F40W/2FT/350BL) to encourage flight, and the measurement temperature was kept constant using an electric heater (mean = 30.3°C, SD = 0.34°C). The total amount of CO_2_ emitted during seven min of flight was used as a measure of FMR. This measure is expected to represent the maximal flight performance of an individual butterfly during seven min of sustained flight. Metabolic rate generally scales positively with body mass, and in intraspecific comparisons, this effect should be accounted for (e.g., Kleiber 1947). To remove the effect of body mass on FMR and enable the examination of mass‐independent FMR differences, the residual from a linear model of FMR against body mass was used, calculated separately for females and males (*R* ^2^ = 0.19, *P* = 0.004 and *R* ^2^ = 0.01, *P *=* *0.228, respectively). Butterflies were allowed to recover from the metabolic measurement for a minimum of 20 h (1–6 days) in 40 × 50 cm mesh cages under favorable conditions (08–10 light/24°C, 10–15 light/28°C, 15–17 light/24°C, 17–08 dark/18°C, 20% honey–water solution ad libitum).

### Flight experiments and thermal imaging

The flight experiments were carried out in mid‐May in semi‐natural conditions in a large outdoor population cage (32 m × 26 m × 3 m; Hanski et al. 2006). The cage is covered with a mesh that prevents butterflies from escaping but allows close to natural environmental conditions (Hanski et al. 2006). Flight experiments were conducted over 5 days during which weather conditions were generally suitable for butterfly flight, although conditions varied within and between measurements. Ambient air temperature at ~1 m above ground, dew point, RH, and solar radiation intensity were recorded at five min intervals with a weather station data logger (HOBO H21‐001; Onset, Bourne, MA) placed inside the population cage. The level of sunshine and windiness were recorded separately using a manual scale from 0 (no clouds/no wind) to 5 (completely overcast/very windy).

Variation in the environmental conditions during the flight experiments was summarized into PCs (principal components) using the prcomp function in R (R Development Core Team 2009). Four original variables were included in the PCA: ambient air temperature, RH, windiness, and the level of sunshine. PC1 explained about 60% of total variance and correlated positively with ambient air temperature (which varied between 12.5 and 20.5°C during the experiments) and sunshine and negatively with RH and windiness (Table 1). PC2 explained about 30% of total variance and correlated most highly and positively with RH. PC3 accounted for most of the remaining variance (~8%), correlating negatively with sunshine and windiness, and it thus describes sunny but windy weather. The first three PCs were included in models explaining thorax takeoff *T*_b_ and thorax cooling during flight.

**Table 1:** Principal components (PCs) that summarize variation in weather conditions during the flight experiments. The weather variables include ambient air temperature (°C), relative humidity (RH), sunshine, and windiness. The table gives correlations of the original variables with the PCs. The header row gives the eigenvalue and percentage of variance explained by each PC, respectively

Weather variable	PC1 (1.543, 59.5%)	PC2 (1.052, 27.7%)	PC3 (0.563, 7.9%)
Ambient air temperature	0.582	−0.245	0.278
RH	−0.299	0.809	0.319
Sunshine	0.501	0.458	−0.734
Windiness	−0.567	−0.275	−0.531

Butterflies were allowed to feed in the morning of the measurement day. Before the flight experiment, butterflies were kept for 10–60 min in 5 × 20 cm cylindrical net cages inside a transparent plastic box in cool temperature. A DIAS PYROVIEW 380L compact (DIAS Infrared GmbH, Dresden, Germany) IR thermal image camera was used to photograph butterflies to measure thorax surface temperature. Surface temperature was measured to allow natural flight behavior during the experiments. It is important to note that the surface temperature may overestimate inner thoracic temperature at flight takeoff (surface warming by solar radiation) and underestimate it following flight (surface cooling due to convection). However, outer and inner thorax temperatures are expected to correlate similarly between different individuals, at least within genders. Differences in, for example, melanization or “fur” thickness could potentially affect the thorax surface–inner thorax temperature ratio, but no significant variation in such traits have been observed in the Glanville fritillary (the Åland population). Finally, the main purpose of the study was not to measure absolute body temperatures, but to compare temperature measurements between individuals with differing flight metabolic rates and genotypes at candidate loci. For the previous reasons, these comparisons are expected to be conservative (see also Saastamoinen and Hanski 2008).

To start the experiment, the butterfly was placed on a platform covered with white cardboard at the height of 50 cm, with the IR camera on a tripod stand focused on the butterfly. Windshields were erected on two sides of the platform. The basking butterfly was photographed at 1‐sec intervals, and the butterfly was allowed to bask until it took off on its own. The flying butterfly was followed on foot, recording the time in flight with a stopwatch. The flight of the Glanville fritillary typically consists of short flight bouts. In the experiment of Ovaskainen et al. (2008) on freely flying butterflies followed with a harmonic radar, the average distance travelled during a flight bout was 32 m. In the present experiment, the butterfly was allowed to either to land on its own (*n *=* *65 flight experiments) or in order to measure changes in *T*_b_ during longer flights, the butterfly was chased to continue its flight immediately after landing (*n* = 103). The average duration of natural (nondisturbed) flight bouts was 9.1 sec (SD = 5.32 sec), whereas the chasing resulted in the average flight time of 16.4 sec (SD = 9.12 sec; the short time during which the butterfly was on the ground is excluded). Results on *T*_b_ were not affected by the chasing action itself (*P*_females_ = 0.421, *P*_males_ = 0.332), but only by the resulting longer flight duration (Results). At the end of the experiment, the butterfly was caught in mid‐flight or immediately after landing and brought back to the focus of the IR camera within 15 sec on average (SD = 7.97 sec) to record body temperature after flight. After the experiment, the butterfly was placed into a net cage in the shade to prevent activity. Most individuals (*n* = 81/87) participated in two flight experiments. The second measurements were performed later on during the same day, after allowing the butterfly to rest for several hours, or after 1–3 days (kept in favorable laboratory conditions with food) depending on the prevailing weather conditions. The average age of butterflies during the first and the second flight experiments was 5 and 6 days, respectively. After completing the second flight experiment, butterflies were preserved in Eppendorf tubes in −20°C for subsequent genetic analysis.

### Genotyping

DNA was extracted from whole thorax samples with NucleoSpin 96 Tissue Core Kit (Macherey‐Nagel GmbH & Co. KG, Düren, Germany). Prior to DNA extraction, the tissue was homogenized by shaking the tissue sample with tungsten beads (Tungsten Carbide Beads, 3 mm; Qiagen, Hilden, Germany) for 90 sec (30 Hz) in a TissueLyser (Qiagen). Cells were lyced overnight in 56°C. DNA extraction was performed according to manufacturer's protocol, excepting centrifuge speed, for which 1500 g was used. The quality of the extraction (to rule out degradation of the DNA) was checked by agarose gel electrophoresis. The DNA concentration was measured with Quant‐iT DNA BR kit (Thermo Fisher Scientific, Waltham, MA) and a TECAN plate reader (Tecan Group Ltd., Männedorf, Switzerland). The concentration of each sample was equalized to a 10 ng/*μ*L solution, of which 10 *μ*L was used for genotyping. The candidate genes and SNPs were selected on the basis of previous association and expression studies on the Glanville fritillary (see de Jong et al. 2014 for a description of the SNPs and their selection criteria). A random subset of the samples (*n*_female_ = 25, *n*_male_ = 30) was genotyped for 14 SNPs in six genes, which included *phosphoglucose isomerase* SNPs *Pgi:331* (also referred to as *Pgi_111* in previous studies), *Pgi:105* and *Pgi:1083*,* flightin* SNP *fln:113*,* glucose‐6‐phosphate 1‐dehydrogenase* SNP *G6p1d:239*,* heat‐shock protein* SNPs *Hsp70_1:206*,* Hsp70_1:134*,* Hsp70_2:100*,* Hsp70_3:71*,* Hsp70_4:166,* and *Hsp70_4:268*,* succinate dehydrogenase complex subunit D* SNP *SDHD:149,* and *troponin‐T* SNPs *TnT1:95* and TnT2:100. Genotyping was performed using Sequenom iPLEX Gold chemistry (Sequenom Inc., San Diego, CA), validated for seven independent samples by direct genomic sequencing with ABI 3730 platform (Life Technologies) according to the manufacturer's protocols. Genotypes were manually validated by visual inspection of peak heights, as well as checked for variability. *Hsp70_3:71* and *TnT1:95* were not variable and were thus excluded from the analyses. Additionally, *Hsp70_4:166 and Hsp70_4:268* were 100% linked, and only *Hsp70_4:166* was included in the analyses, resulting in a set of 11 SNPs in six genes.

### Genotype–FMR associations

Associations of FMR with the 11 SNPs were analyzed with ANOVA (analysis of variance) (Table S1, Supporting information). In the case of *Pgi:331* and *Hsp70_1:206*, all three SNP genotypes were analyzed separately and with the rare homozygotes (CC in *Pgi*, GG in *Hsp70*) pooled with the heterozygotes. To balance between the avoidance of type I and type II errors, both uncorrected *P*‐values and *P*‐values corrected for multiple testing (FDR, false discovery rate correction) were calculated. Three SNPs (*Pgi:331*,* fln:113,* and *Hsp70_1:206*) had a significant association with FMR (Table S1 and Results) and were hence chosen for further analyses in models of body temperature.

### Analysis of thermal image data

The images from the thermal image camera were analyzed using the PYROSOFT Compact software (DIAS Infrared Systems, Dresden, Germany). Emissivity of 0.95 was used based on common emissivity of a dark matte surface such as the butterfly thorax. An automatically adjusting thermal scale was used to maximize resolution of the IR thermal images. The last image of the basking butterfly before takeoff and the first image of the butterfly after flight were analyzed to obtain thoracic temperature (*T*_b_) at takeoff and after flight, respectively. The average surface temperature at five random points within the outline of the thorax was used as a measure of thoracic temperature.

### Statistical analyses

Two measures of body temperature were used, the takeoff thorax temperature (C°; takeoff *T*_b_) and the extent of thorax cooling during flight, which was calculated as the difference between postflight *T*_b_ and takeoff *T*_b_ (C°; Δ). One obvious outlier observation with a large negative cooling value was excluded from the data for males. All variables were checked for normality, and flight duration was log‐transformed. Factors affecting takeoff *T*_b_ and Δ were modeled with linear mixed‐effects models using the R package nlme (Pinheiro et al. 2013), with individual identity as a random factor, and using mass‐corrected FMR (residual) as a measure of flight metabolic rate. In models including both genders, many variables involved significant interactions with sex (see Table S2). To facilitate biological interpretation, females and males were subsequently analyzed separately. The effects of the following explanatory variables on takeoff *T*_b_ and Δ were tested: FMR, adult body mass, age, weather variables (PCs, see above), and their interactions with FMR. The PCs explained takeoff *T*_b_ and Δ better than the original weather variables. The model explaining Δ included additionally flight duration (log‐transformed). Only the actual flight time is included in flight duration, and the measurement delay (time between landing and temperature recording) and the time between flight bouts are assumed to vary randomly. Nonsignificant interactions were omitted from the models in a stepwise reduction procedure. Finally, the effects of three FMR‐associated SNP genotypes (in the *Pgi*,* Hsp70* and *flightin* genes, see above) on takeoff *T*_b_ and Δ were analyzed by replacing FMR in the models with the SNP genotype.

Individual‐level repeatability of takeoff *T*_b_ and cooling (∆) based on repeated measurements (*n*_males_ = 38, *n*_females_ = 35; in Tables 3 and 4) were estimated using a linear mixed model‐based (where individual identity is included as a random effect) repeatability method (Nakagawa and Schielzeth 2010). Repeatability (*R*) was calculated as follows:

$$R=\frac{\sigma_{\alpha }^{2}}{\sigma_{\alpha }^{2}+\sigma_{\epsilon }^{2}},$$

where $\sigma_{\alpha }^{2}$ and $\sigma_{\epsilon }^{2}$ are the between‐group and residual (within‐group) variances, respectively. Statistical significance of *R* was estimated based on the ML (maximum likelihood) of the full model and the ML of a model without the random factor (null model). The test statistic is calculated as 2*(ML_full model_ − ML_null model_), and it follows the *χ* ^2^‐distribution with one degree of freedom.

## Results

### Body temperature during flight

**Figure 1:**
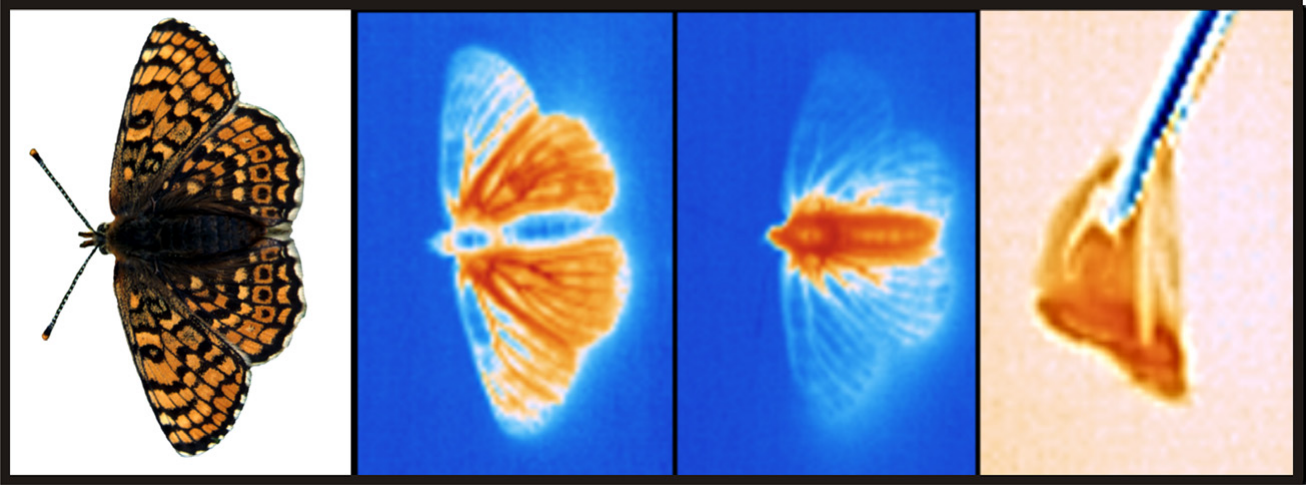
Photograph and infrared thermal images of the Glanville fritillary butterfly (*Melitaea cinxia*). A butterfly (from the second image from left) in the beginning of basking, right before takeoff, and right after capture. The colors represent relative temperature (blue = cold, red = warm). Photograph: Tari Haahtela.

Butterflies basked for 1–10 min before taking off on their own. Figure 1 shows an example of IR thermal images of butterflies in the beginning of basking, at the time of takeoff, and immediately after flight. Table 2 shows summary statistics for the body temperature measurements. The mixed‐effects models for factors affecting thorax takeoff *T*_b_ and cooling (∆) during flight, with butterfly individual as a random factor, are shown in Tables 3 and 4, respectively.

**Table 2:** Summary statistics of body mass and thermal parameters. The statistics include sample size (*n*), minimum (Min.), maximum (Max.), mean, and standard deviation (SD) values, separately for females and males

Trait	Females					Males				
	*n*	Min.	Max.	Mean	SD	*n*	Min.	Max.	Mean	SD
Body mass (mg)	36	59	124	96	15.5	52	43	71	55	6.5
Takeoff temp. (°C)	71	23.0	40.5	31.9	3.82	87	20.4	39.6	31.0	5.06
Temp. after flight (°C)	71	19.8	34.4	27.9	3.02	97	18.2	36.3	27.0	3.75
Cooling (∆; °C)	71	−1.9	11.1	4.0	2.52	87	−2.4	12.9	3.5	2.99
Cooling rate (°C/sec)	71	−0.15	1.42	0.37	0.30	87	−0.50	0.96	0.24	0.25

**Table 3:** Linear mixed‐effects model of butterfly thorax *T*_b_ (°C) at the time of takeoff. Results are shown separately for females (*n*_females_ = 36, *n*observations = 71) and males (*n*_males_ = 49, *n*_observations_ = 87)

Takeoff *T* (°C)	Females					Males				
	Value	Std. error	df	*t*‐Value	*P*	Value	Std. error	df	*t*‐Value	*P*
Adult mass	−0.029	0.030	33	−0.962	0.343	0.153	0.074	46	2.074	**0.044**
Int. rate of flight metabolism (residual)	0.083	0.460	33	0.180	0.858	−0.667	0.490	46	−1.362	0.180
Weather PC 1	0.206	0.274	32	0.749	0.459	1.695	0.557	35	3.042	**0.004**
Weather PC 2	−0.548	0.485	32	−1.131	0.267	−0.700	0.505	35	−1.387	0.174
Weather PC 3	1.027	0.783	32	1.313	0.199	−2.144	1.013	35	−2.117	**0.041**

Statistically significant effects are shown in bold.

**Table 4:** Linear mixed‐effects model of butterfly thorax cooling (∆; °C) during flight for females (*n*_females_ = 36, *n*_observations_ = 71) and males (*n*_males_ = 49, *n*_observations_ = 85). ∆ is calculated as the difference between thorax *T*_b_ at the time of takeoff and after landing from flight

Cooling (°C)	Females					Males				
	Value	Std. error	df	*t*‐Value	*P*	Value	Std. error	df	*t*‐Value	*P*
Flight duration (log)	1.969	0.345	31	5.706	**0.000**	2.837	0.371	32	7.650	**0.000**
Adult mass	−0.035	0.014	33	−2.414	**0.022**	0.006	0.040	46	0.151	0.881
Int. rate of flight metabolism (residual)	0.087	0.222	33	0.392	0.698	−0.774	0.270	46	−2.863	**0.006**
Weather PC 1	−0.777	0.140	31	−5.561	**0.000**	−0.425	0.278	32	−1.530	0.136
Weather PC 2	0.704	0.237	31	2.965	**0.006**	−0.544	0.248	32	−2.198	**0.035**
Weather PC 3	−0.140	0.384	31	−0.365	0.717	−1.552	0.498	32	−3.115	**0.004**

Statistically significant effects are shown in bold.

**Figure 2:**
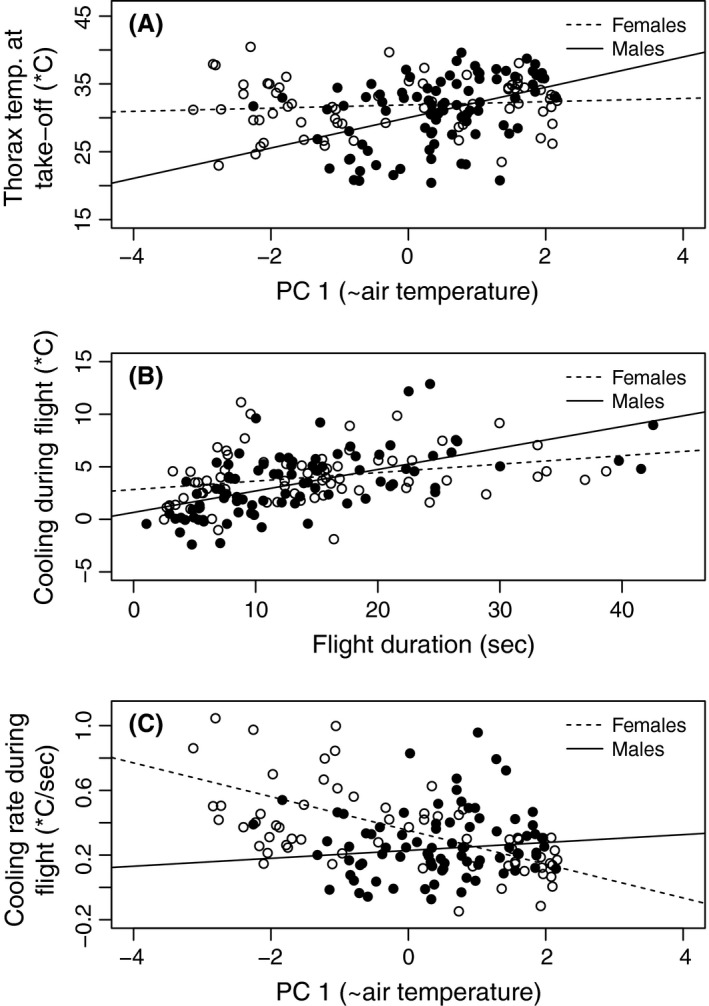
Butterfly body temperature measures in relation to environmental conditions and the duration of flight. (A) Thorax *T*_b_ (°C) at takeoff in relation to weather PC 1 (~air temperature) (*P*_females_ = 0.378, *P*_males_ = 4.3e‐05). (B) Thorax *T*_b_ cooling (∆; °C) in relation to flight duration (sec; *P*_females_ = 0.0156, *P*_males_ = 4.03e‐09). ∆ is calculated as the difference between thorax *T*_b_ at the time of takeoff and after landing. (C) Thorax cooling rate (∆/sec) in relation to weather PC 1 (~air temperature) (°C; *P*_females_ = 3.8e‐08, *P*_males_ = 0.384). Results are shown for females (open circles, dotted line) and males (black circles, black line).

**Figure 3:**
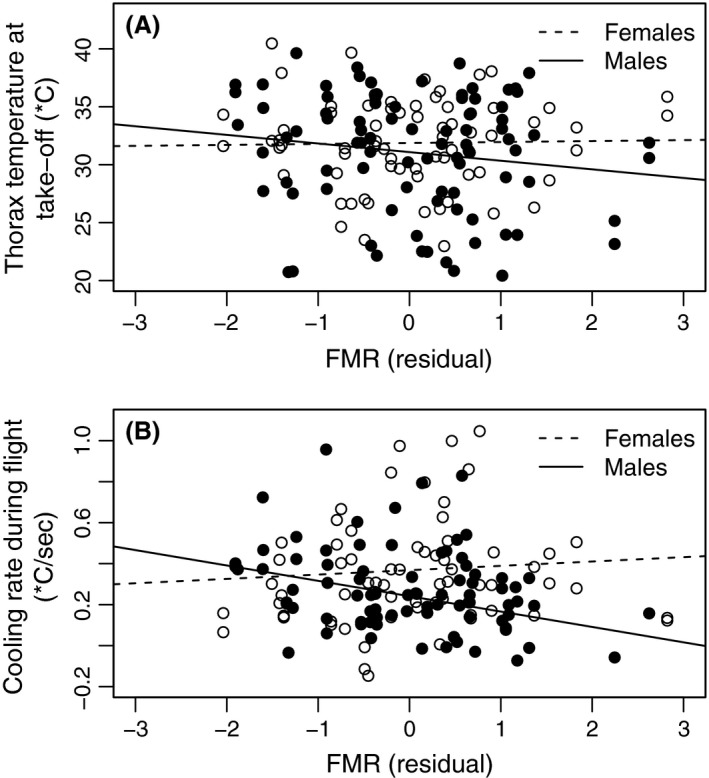
Butterfly body temperature measures in relation to flight metabolic rate (FMR). (A) Thorax *T*_b_ (°C) at takeoff (*P*_females_ = 0.856, $R_{\mathrm{females}}^{2}$ = 0, *P*_males_ = 0.162, $R_{\mathrm{males}}^{2}$ = 0.01141) and (B) thorax cooling rate (∆/sec; *P*_females_ = 0.552, $R_{\mathrm{females}}^{2}$ = 0, *P*_males_ = 0.00494, $R_{\mathrm{males}}^{2}$ = 0.08038) in relation to (FMR). Cooling rate ∆ is calculated by dividing takeoff *T*_b_ – flight *T*_b_ by flight duration (sec), and FMR is the residual from a linear model of FMR against adult mass. Results are shown for females (open circles, dotted line) and males (black circles, black line).

Thorax *T*_b_ at takeoff varied from 23.0 to 40.5°C in females (average 31.9°C, SD 3.82°C) and between 20.4 and 39.6°C in males (average 31.0°C, SD 5.06°C; Table 2). There were no significant effects of environmental or other sources of variation on female takeoff *T*_b_. PC1 (related to ambient air temperature) was positively correlated with takeoff *T*_b_ in males (*P *=* *0.004) but not in females (*P *=* *0.459; Table 3, Fig. 2A; *P *=* *0.017 for the sex – PC1 interaction; Table S2). In addition to the effect of PC1, male takeoff *T*_b_ was significantly affected by butterfly mass, with large males taking off at a significantly lower *T*_b_ than small males (*P *=* *0.044; for details on body mass, see Table 2). Flight metabolic rate was not significantly associated with takeoff *T*_b_ in males (*P *=* *0.180, Table 3; Fig. 3A).

Butterflies cooled down significantly during flight, the more the longer the flight (Fig. 2B). Females cooled down during flight with an average rate of 0.37°C/sec (SD 0.30°C/sec), compared to 0.24°C/sec in males (SD 0.25°C/sec; Table 2). The faster cooling rate of females (54% faster, *P *=* *0.043; Table S2, see also Fig. 2C) may be partly explained by exceptionally low ambient temperatures experienced by some females (16 and 4 flight experiments were conducted in air temperatures below 14°C in females and males, respectively). Cooling was strongly and positively affected by the duration of the flight in both sexes (Fig. 2B, Table 4). The effect appeared to be less in females than in males (*P*_females_ = 0.016, *P*_males_ = 4.03e‐09), but the sex–flight duration interaction was not statistically significant (*P* = 0.115 for, Table S2). Otherwise different factors affected cooling in males and females (Table 4, Table S2). In females but not in males, cooling was negatively affected by PC1 (related to air temperature; *P*_females_ < 0.001 and *P*_males_ = 0.136, *P* = 0.357 for the sex–PC1 interaction; Table 4, Fig. 2C). PC2 (related to humidity) affected cooling positively in females (*P* = 0.006), but negatively in males (*P* = 0.035). Additionally, PC3 (related to windiness) significantly affected cooling in males (*P* = 0.004). Body mass appeared to have an effect on cooling in females only, with larger females cooling less (*P* = 0.022). Finally, flight metabolic rate had a strong and significant negative effect on cooling during the flight in males (*P* = 0.006; Fig. 3B) but not in females (*P* = 0.698; *P* = 0.014 for the sex–FMR interaction, Table S2). Considering male butterflies with higher versus lower FMR than the average, the average rate of cooling was 0.29°C/sec for the low‐FMR males and 0.19°C/sec for high‐FMR males. Thus, during 30 sec of flight, males with low FMR cooled down, on average, 8.7°C, whereas males with high FMR cooled down only 5.7°C.

Thorax *T*_b_ at takeoff and thorax cooling during the flight had low and nonsignificant repeatability in males (*R* = 0.047, *P *>* *0.05; *R* = 0.293, *P *>* *0.05, respectively). In females, the measures of takeoff *T*_b_ and cooling rate were clearly not repeatable (*R* = 5.5e‐09, *P *>* *0.05; *R* = 4.8e‐09, *P *>* *0.05, respectively).

### The association of SNP genotypes with flight metabolic rate and body temperature during flight

The results of the association analyses are given in Table S1. In the case of SNPs with a significant association with FMR, I examined the corresponding associations with the body temperature measurements related to flight. These SNPs are in the genes *phosphoglucose isomerase* (SNP *Pgi:*331), *heat‐shock protein 70 kDa* (SNP *Hsp70_1:206*), and *flightin* (SNP *fln:113*).

**Figure 4:**
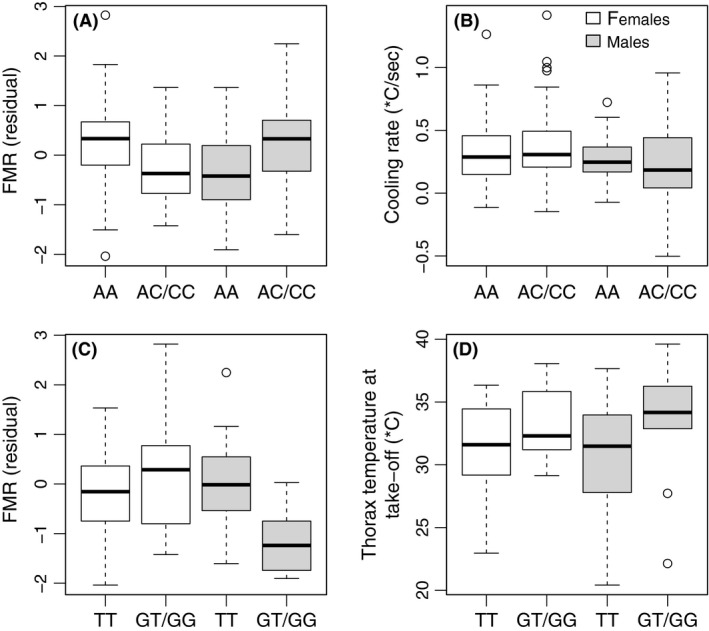
Association of *Pgi* and *Hsp70* genotypes with flight metabolic rate (FMR) and body temperature measures. (A) Association of *Pgi* genotype with FMR. (B) Association of *Pgi* genotype (SNP *Pgi:331*) with thorax *T*_b_ cooling during flight (∆; °C). (C) Association of *Hsp70* genotype (SNP *Hsp70_1:206*) with FMR. (D) Association of *Hsp70* genotype (SNP *Hsp70_1:206*) with thorax takeoff *T*_b_ (°C). Results are shown for females (white) and males (gray).

In the case of *Pgi:331*, there was a significant sex–genotype interaction (*P* = 0.009), such that the pooled SNP genotypes AC/CC were associated with high FMR in males (*P* = 0.050) but not in females (Fig. 4A). The *Pgi* genotypes did not significantly differ in cooling rate during flight (Δ/sec) (linear mixed model excluding weather effects; Fig. 4B). In a linear mixed model of Δ explained by the weather PCs (model as in Table 4, but FMR replaced by *Pgi* genotype), *Pgi* genotype had no effect in either sex (*P*_males_ = 0.803, *P*_females_ = 0.359). *Pgi* was not associated with takeoff *T*_b_.

The SNP genotype TT in *Hsp70_1:206* was associated with elevated FMR in males but not in females (Fig. 4C; *P*_males_ = 0.004, $R_{\mathrm{males}}^{2}$ = 0.238, *P*_females_ = 0.4458, $R_{\mathrm{females}}^{2}$ = 0, in males, the association remained significant after correcting for multiple testing, FDR = 0.048; *P *=* *0.0122 for the sex–*Hsp70* genotype interaction). The same *Hsp70* SNP genotype was also associated with reduced takeoff *T*_b,_ similarly in both sexes (Fig. 4D; *P *=* *0.0287; linear mixed model for both sexes as in Table 3, but with FMR replaced by *Hsp70* genotype and sex included as a factor). In the *flightin* gene (SNP *fln:113*), the association with FMR was weak in males and nonsignificant in females (*P*_males_ = 0.026, *P*_females_ = 0.730), and there were no significant associations with the measures of body temperature.

## Discussion

### Thermal tolerance and takeoff temperature

The body temperature of basking butterflies increases rapidly above the ambient air temperature due to solar radiation, and there is even a risk of overheating, which can result in reduced survival and fecundity (Rawlins 1980; Kingsolver and Watt 1983). For example, *Colias* butterflies cease flight activity and behaviorally avoid further heating when *T*_b_ exceeds 40–42°C (Kingsolver and Watt 1983). In the present study, the thoraces of basking butterflies reached surface temperatures as high as 40.5°C, which is likely to be close to the upper thermal tolerance limit. In this context, it is noteworthy that the strongest effect on takeoff *T*_b_ apart from the external factors was allelic variation in a SNP in the *heat‐shock 70‐kDa protein* (*Hsp70*) locus. *Hsp*s are upregulated in response to environmental stressors, and they are important in protecting against cellular damage, especially those caused by exposure to extreme temperatures (Sorensen et al. 2003). Here, *Hsp70* SNP *Hsp70_1:206* genotype TT was associated with significantly reduced takeoff *T*_b_ both in females and males. This result suggests that butterflies with this genotype are more susceptible to overheating and cannot allow takeoff *T*_b_ to reach values as high as butterflies with the other genotypes. Because the heating of the butterfly during basking is mostly based on solar radiation (Wickman 2009), avoidance of overheating may be an important factor affecting flight takeoff behavior even at northern latitudes. To better understand the association of *Hsp70* genotype with flight thermal dynamics, future studies should address the relationship between SNP genotype and *Hsp70* expression levels.

Previous studies have suggested that variation in *Hsp70* expression can buffer individual differences in thermal tolerance (Rutherford 2003). In the willow beetle *C. aeneicollis*, genetic variation in *Pgi* is associated with dissimilar expression of *Hsp70* in response to thermal stress (Dahlhoff and Rank 2000; Neargarder et al. 2003; McMillan et al. 2005). *Pgi* genotype is known to influence tolerance of extreme temperatures (Watt et al. 1983; Dahlhoff and Rank 2000; Neargarder et al. 2003; Rank et al. 2007; Luo et al. 2014).Thus, the less thermally tolerant *Pgi* genotypes upregulate *Hsp70* to a greater extent (Dahlhoff and Rank 2000; Rank et al. 2007). In the Glanville fritillary, individuals with the *Pgi* genotype associated with high FMR in standard temperatures do worse as temperatures increase (Niitepõld 2010). Here, the same *Hsp70* SNP (*Hsp70_1:206*) which influenced takeoff *T*_b_ was also significantly associated with FMR in male butterflies, explaining as much as 24% of variation in FMR. The *Hsp70* genotype associated with high FMR (and expected low thermal tolerance) had reduced takeoff *T*_b_, suggesting that *Hsp70* genotype, thermal tolerance, and flight takeoff behavior may be causally connected.

### Body temperature at flight is affected by flight metabolic rate

Rate of flight metabolism had a highly significant effect on cooling rate during flight in male butterflies, despite their small size. Low‐FMR males cooled down about 1.5 times faster during flight than males with high FMR. In contrast, FMR had no effect on *T*_b_ in females (discussed in the next section). In small butterflies, endothermic heating due to flight metabolism has been commonly presumed to have a negligible effect on flight compared to external sources of heat (Shreeve 1984; Heinrich 1986b; Wickman 2009), while in large butterflies, the heating of flight muscles by metabolism has been demonstrated (Heinrich 1986a,b; Tsuji et al. 1986). Intraspecific variation in flight metabolism and its connection with flight thermal dynamics has not been previously studied. In the present study, measurements were conducted in near natural conditions encompassing a natural range of environmental variation. The observed variation among individuals during a single short flight bout can be expected to have significant consequences for the fitness and dispersal distances in the life time of a butterfly, during which it performs thousands of such short flight bouts (see Ovaskainen et al. 2008).

The relationship between cooling during flight and FMR could, in principle, be explained by FMR‐dependent differences in takeoff *T*_b_. This is because taking off at low *T*_b_ leads to a smaller absolute difference between body temperature and the ambient air temperature, which would decrease the rate of cooling. However, in the present results, FMR does not have a significant effect on takeoff *T*_b_. The weak and statistically nonsignificant negative trend between takeoff *T*_b_ and FMR (Table 3, Fig. 3A) could reflect the wider range of behavioral options for butterflies with high flight metabolism, which would have sufficient time for the flight bout even if body temperature at takeoff would be relatively low. In contrast, low‐FMR individuals taking off at similar low *T*_b_ would be forced to land soon after takeoff due to their faster cooling, and therefore, they would need to “buy” more time (see also Heinrich 1986a,b) by attaining higher takeoff *T*_b_. Ovaskainen et al. (2008) showed that the longer dispersal distances at low ambient temperatures of butterflies from newly colonized populations, which consist of dispersive individuals with higher than average FMR (Hanski et al. 2002; Haag et al. 2005; Hanski and Mononen 2011), were not due to longer individual flight bouts but to their higher frequency. This suggests that the more active individuals take off at cooler body temperature but can still perform flight bouts of average duration.

Previous work on the Glanville fritillary has shown that butterflies with the *Pgi* genotype associated with high FMR and dispersal rate tend to have higher than average *T*_b_ during flight (Saastamoinen and Hanski 2008). In the present study, male butterflies with different *Pgi* genotypes differed on average 18% in FMR (*Pgi:331* AC/CC vs. AA), which is similar to the 17% difference found by Haag et al. (2005), while other studies have reported even greater differences (Niitepõld et al. 2009; Niitepõld 2010). Variation in the results may be due to acclimatization to different thermal conditions prior to the experiments, as the differences between the *Pgi* genotypes appear to be greatest in individuals acclimatized to low ambient temperatures (S.C. Wong, A. Oksanen, A.L.K. Mattila, K. Niitepõld, R. Lehtonen, and I. Hanski. unpubl. data). In the present study, males with the AC and CC genotypes (with higher FMR) appeared to cool down at a somewhat lower rate than the AA individuals (Fig. 4A and B), but the difference was not significant. In the study of Niitepõld et al. (2009), *Pgi* genotype had no significant effect on the probability of flight activity within a short period of time, but individuals with high FMR were significantly more active and less likely to stop flying than low‐FMR butterflies. These results suggest that while *Pgi* genotype, FMR and body temperature at flight are all correlated, body temperature at flight is causally affected by FMR rather than by *Pgi* genotype.

### Sex differences in flight thermal dynamics

The dynamics of body temperature at flight and factors affecting it were significantly different between the two sexes, and the above discussion applies primarily to males. The contrasting results for the two sexes are best explained by differences in body mass and differential allocation to different body parts in females and males (Gilchrist 1990). Females are significantly heavier than males (here 75% heavier; Table 2), and they allocate most of their mass to the abdomen rather than to flight muscles in the thorax (see also Saastamoinen et al. 2009). Females had on average higher thorax *T*_b_, consistent with other studies on butterflies (Pivnick and McNeil 1986; Gilchrist 1990; Saastamoinen and Hanski 2008). Females have greater wing loading (body mass/wing area; around 35% greater in the Glanville fritillary; Mattila et al. 2012), which is expected to require higher wing‐beat frequency and thus higher *T*_b_ (Heinrich 1974; Pivnick and McNeil 1986). This may make females more constrained by environmental conditions, that is, they may be able to be active under a narrower thermal window than males (Gilchrist 1990). The size of the thermal window for flight is expected to be especially important for females, with a direct influence on reproductive success (Kingsolver 1983; Watt 1992; Saastamoinen and Hanski 2008). On the other hand, smaller butterflies (males) are more susceptible to convective cooling due to their greater surface area‐to‐volume ratio (Gilchrist 1990). In the tropical butterfly *Bicyclus anynana*, the flight activity of males is more influenced by environmental conditions than that of the larger females (Saastamoinen et al. 2012). In sum, the flight of females may be more restricted by attaining suitable takeoff body temperature, whereas the flight of males by maintaining body temperature when already in flight.

In the present study, body mass affected thermal dynamics but in a dissimilar manner in females and males. Smaller males took off with lower *T*_b_, but no such effect was found in females. As the only significant factor affecting female takeoff *T*_b_ was *Hsp70* genotype, it may be that the unmated females used in this study lacked motivation to fly, besides avoiding overheating (Rawlins 1980; Kingsolver and Watt 1983). Flight motivation can be assumed to be governed by different factors in females and males, for which the function of flight differs greatly (Niitepõld et al. 2011). In short, females fly to find suitable oviposition sites (once mated), while males fly to keep a mating territory and to look for mates (both “perching” and “patrolling” male mate‐location strategies are observed in the Glanville fritillary; Boggs and Nieminen 2004).

The above‐mentioned gender differences in flight motivation (potentially affecting natural flight behavior of females in the experimental conditions) could partly explain why FMR only affected cooling during flight in males but not in females. A possible gender difference between surface versus inner thorax temperature ratio and the low ambient temperatures experienced by some females could be potential sources of bias when comparing the sexes, but the on average higher takeoff *T*_b_ in females compared with males suggests against the latter. However, a plausible biological explanation is that because males have markedly higher FMR per unit of body mass than females (here, 79% higher), also the heating of flight muscles caused by FMR is greater in males, which may override the effect of faster convective cooling due to greater surface area‐to‐volume ratio. Also, the difference between *T*_b_ and ambient air temperature is greater in females, because of their higher takeoff *T*_b_. These hypotheses are consistent with the observed higher rate of cooling in females than males (54% difference).

## Conclusions

Contrary to what is commonly expected for small butterflies, flight metabolic rate significantly influenced the dynamics of body temperature during flight in male Glanville fritillaries, with likely consequences for fitness and dispersal in varying environmental conditions. The results also suggest that the tolerance of high temperatures may be another important factor influencing flight capacity in butterflies and other similar insects. This study has highlighted the extent of intraspecific variation in dispersal‐related thermal performance. Such knowledge of the physiological performance of insects in different thermal environments is needed for predictive models of the evolution of dispersal in the face of habitat fragmentation and climate change (Helmuth et al. 2005).

## Data accessibility

Data will be made available in the Dryad repository (http://datadryad.org/) on acceptance for publication.

## Conflict of Interest

The author declares no conflict of interests.

## Supporting information


**Table S1.** The associations of SNP genotypes at candidate loci with flight metabolic rate.
**Table S2.** Linear mixed‐effects models of butterfly body temperature measures with all sex interaction terms.
