# Supplementary material for: Thermal biology of flight in a butterfly: genotype, flight metabolism, and environmental conditions
Source: Ecol Evol. 2015 Nov 10;5(23):5539–51. doi: 10.1002/ece3.1758 (PMC4813115; doi:10.1002/ece3.1758)
Supplement: Supplementary file 1 — Table S1. The associations of SNP genotypes at candidate loci with flight metabolic rate. Table S2. Linear mixed‐effects models of butterfly body temperature measures with all sex interaction terms. [file ECE3-5-5539-s001.docx]

**Thermal biology of flight in a butterfly: genotype, flight metabolism and environmental conditions**

Anniina L. K. Mattila

Metapopulation Research Group, Department of Biosciences, University of Helsinki, FI-00014 Helsinki, Finland

[anniina.mattila@helsinki.fi](mailto:anniina.mattila@helsinki.fi)

**Supporting information**

**Table S1.** The associations of SNP genotypes at candidate loci with flight metabolic rate. The table gives the results for ANOVA (*F*- and *P*- values) as well as statistical significance after correcting for multiple testing (false discovery rate, FDR correction). Significant associations and the SNPs chosen for further analysis in models of body temperature are shown in bold. *Pgi:331* and *Hsp70_1:206* were analyzed with all genotypes separately and with the minor homozygote genotype pooled with the heterozygote genotype.

|  |  |  |  |  |  |  |  |
| --- | --- | --- | --- | --- | --- | --- | --- |
|  |  |  |  |  |  |  |  |
|  | Females | | |  | Males | | |
| Gene (SNP) | *F* | *P* | *FDR* |  | *F* | *P* | *FDR* |
| *Pgi* (*Pgi:331* = *Pgi_AA111*) | 1.505 | 0.237 | 0.770 |  | 2.005 | 0.146 | 0.272 |
| ***Pgi* (*Pgi:331* = *Pgi_AA111*) CC+CA** | 3.098 | 0.087 | 0.684 |  | 4.041 | **0.050** | 0.163 |
| *Pgi* (*Pgi:105*) | 1.157 | 0.327 | 0.850 |  | 2.468 | 0.096 | 0.249 |
| *Pgi* (*Pgi:1083*) | 1.964 | 0.158 | 0.684 |  | 0.553 | 0.579 | 0.837 |
| *SDHD* (*SDHD:149*) | 0.009 | 0.925 | 0.931 |  | 0.700 | 0.411 | 0.668 |
| ***flightin* (*fln:113*)** | 0.122 | 0.730 | 0.931 |  | 4.214 | **0.026** | 0.111 |
| *glucose-6-phosphate* (*G6p1d:239*) | 0.571 | 0.573 | 0.931 |  | 0.124 | 0.884 | 0.884 |
| *Hsp70* (*Hsp70_1:206*) | 0.297 | 0.746 | 0.931 |  | 5.210 | **0.012** | 0.079 |
| ***Hsp70* (*Hsp70_1:206*) GG+GT** | 0.602 | 0.446 | 0.931 |  | 10.060 | **0.004** | **0.048** |
| *Hsp70* (*Hsp70_1:134*) | 0.346 | 0.563 | 0.931 |  | 0.115 | 0.738 | 0.872 |
| *Hsp70* (*Hsp70_3:71*) | 2.372 | 0.117 | 0.684 |  | 0.326 | 0.725 | 0.872 |
| *Hsp70* (*Hsp70_4:166*) | 0.072 | 0.931 | 0.931 |  | 2.182 | 0.132 | 0.272 |
| *troponin-T* (TnT2:100) | 0.136 | 0.874 | 0.931 |  | 0.218 | 0.806 | 0.873 |
|  |  |  |  |  |  |  |  |
|  |  |  |  |  |  |  |  |

**Table S2.** Linear mixed effects models of butterfly body temperature measures with all sex interaction terms. (**A**) butterfly thorax T_b_ (°C) at the time of take-off and of (**B**) butterfly thorax cooling during flight (∆; °C, the difference between thorax T_b_ at the time of take-off and after landing from flight). The models include both sexes and all sex interaction terms (*n_females_*=36, *n _female observations_*=71; *n_males_*=49, *n_male observations_*=85). Significant effects are shown in bold.

|  |  |  |  |  |
| --- | --- | --- | --- | --- |
| **A) Take-off T_b_ (°C)** |  |  |  |  |
|  | *df* | *t-value* | *P* |  |
| **Sex** | 79 | -2.523 | **0.014** |  |
| Adult mass (mg) | 79 | -0.882 | 0.381 |  |
| Int. FMR (residual) | 79 | 0.165 | 0.869 |  |
| Weather PC 1 | 67 | 0.687 | 0.494 |  |
| Weather PC 2 | 67 | -1.037 | 0.303 |  |
| Weather PC 3 | 67 | 1.204 | 0.233 |  |
| **Sex : Adult mass (mg)** | 79 | 2.410 | **0.018** |  |
| Sex : Int. FMR (residual) | 79 | -1.130 | 0.262 |  |
| **Sex : Weather PC 1** | 67 | 2.443 | **0.017** |  |
| Sex : Weather PC 2 | 67 | -0.204 | 0.839 |  |
| **Sex : Weather PC 3** | 67 | -2.519 | **0.014** |  |
|  |  |  |  |  |
| **B) Cooling (Δ, °C)** |  |  |  |  |
|  |  |  |  |  |
| **Sex** | 79 | -2.053 | **0.043** |  |
| **Flight duration (log)** | 63 | 5.088 | **0.000** |  |
| **Adult mass (mg)** | 79 | -2.099 | **0.039** |  |
| Int. FMR (residual) | 79 | 0.336 | 0.738 |  |
| **Weather PC 1** | 63 | -4.867 | **0.000** |  |
| **Weather PC 2** | 63 | 2.644 | **0.010** |  |
| Weather PC 3 | 63 | -0.230 | 0.819 |  |
| Sex : Flight duration (log) | 63 | 1.599 | 0.115 |  |
| Sex : Adult mass (mg) | 79 | 1.016 | 0.313 |  |
| **Sex : Int. FMR (residual)** | 79 | -2.511 | **0.014** |  |
| Sex : Weather PC 1 | 63 | 0.927 | 0.357 |  |
| **Sex : Weather PC 2** | 63 | -3.499 | **0.001** |  |
| **Sex : Weather PC 3** | 63 | -2.414 | **0.019** |  |
|  |  |  |  |  |
